# Impact of gastroesophageal reflux disease on patients' daily lives: a European observational study in the primary care setting

**DOI:** 10.1186/1477-7525-7-60

**Published:** 2009-07-02

**Authors:** Javier P Gisbert, Alun Cooper, Dimitrios Karagiannis, Jan Hatlebakk, Lars Agréus, Helmut Jablonowski, Javier Zapardiel

**Affiliations:** 1 Department of Gastroenterology, Hospital Universitario de la Princesa, Madrid, Spain, and Centro de Investigación Biomédica en Red de Enfermedades Hepáticas y Digestivas (CIBEREHD), Spain; 2 Bridge Medical Centre, Crawley, West Sussex, UK; 3 Department of Gastroenterology, Athens Medical Center, Athens, Greece; 4 Institute of Medicine, Haukeland University Hospital, Bergen, Norway; 5 Center for Family and Community Medicine, Karolinska Institutet, Huddinge/Stockholm, Sweden; 6 Klinikum Salzgitter GmbH, Salzgitter, Germany; 7 AstraZeneca, Madrid, Spain

## Abstract

**Background:**

The impact of gastroesophageal reflux disease (GERD) on the daily lives of patients managed in primary care is not well known. We report the burden of GERD in a large population of patients managed in primary care, in terms of symptoms and impact on patients' daily lives.

**Methods:**

RANGE (*R*etrospective *AN*alysis of *GE*RD) was an observational study that was conducted at 134 primary care sites across six European countries. All adult subjects who had consulted their primary care physician (PCP) during a 4-month identification period were screened retrospectively and those consulting at least once for GERD-related reasons were identified. From this population, a random sample of patients was selected to enter the study and attended a follow-up appointment, during which the Reflux Disease Questionnaire (RDQ), the GERD Impact Scale (GIS) and an extra-esophageal symptoms questionnaire were self-administered. Based on medical records, data were collected on demographics, history of GERD, its diagnostic work-up and therapy.

**Results:**

Over the 4-month identification period, 373,610 subjects consulted their PCP and 12,815 (3.4%) did so for GERD-related reasons. From 2678 patients interviewed (approximately 75% of whom reported taking medication for GERD symptoms), symptom recurrence following a period of remission was the most common reason for consultation (35%). At the follow-up visit, with regard to RDQ items (score range 0–5, where high score = worse status), mean Heartburn dimension scores ranged from 0.8 (Sweden) to 1.2 (UK) and mean Regurgitation dimension scores ranged from 1.0 (Norway) to 1.4 (Germany). Mean overall GIS scores (range 1–4, where low score = worse status) ranged from 3.3 (Germany) to 3.5 (Spain). With regard to extra-esophageal symptoms, sleep disturbance was common in all countries in terms of both frequency and intensity.

**Conclusion:**

In this large European observational study, GERD was associated with a substantial impact on the daily lives of affected individuals managed in the primary care setting.

## Background

Gastroesophageal reflux disease (GERD) is a chronic condition in which reflux of stomach contents causes troublesome symptoms and/or complications [1]. The disease can present in terms of a range of esophageal and extra-esophageal syndromes, but its cardinal symptoms are heartburn and regurgitation [1]. It is increasingly recognised that such symptoms can be severely detrimental to health-related quality of life (HRQOL), disrupting patients' daily lives in terms of physical, social and emotional well-being [2,3]. Indeed, the negative effect of GERD on HRQOL is becoming better defined as researchers increasingly make use of patient-reported outcome (PRO) instruments to investigate the impact of GERD symptoms in large populations [4]. The German ProGERD study, for example, determined that patients with symptoms of GERD had substantially impaired HRQOL in terms of both physical and psychosocial aspects of well-being compared with the general population, and felt restricted as a result of food and drink problems, disturbed sleep, and impaired vitality and emotional well-being [5]. Studies conducted in the Swedish general population assessing the impact of the severity and frequency of GERD symptoms on HRQOL have found that even symptoms rated as mild are associated with a clinically meaningful reduction in well-being [6], and that weekly symptoms are likely to have a clinically significant adverse impact on most aspects of patients' daily lives [7]. Consequently, the impact of symptoms on patients' daily lives is one of the most common reasons for consultation for GERD [8], which accounts for a significant workload among primary care physicians (PCPs) [9]. However, the impact of symptoms of GERD among patients who are managed and treated in primary care has not been well studied.

As part of the RANGE (*R*etrospective *AN*alysis of *GE*RD) study, which aimed to document the symptom profile, diagnosis and management of GERD patients in several European countries, we evaluated the burden of GERD using a selection of PRO instruments, the results of which are reported in this paper.

## Methods

### Study design and patients

RANGE was a multicentre, multinational, observational study (AstraZeneca study code: D9612L00114) conducted as a series of parallel, locally managed studies at 134 primary care sites across six European countries (Germany, Greece, Norway, Spain, Sweden and the UK). The study was conducted in accordance with the ethical principles described in the Declaration of Helsinki, and was approved by local ethics committees in each country.

**Figure 1:**
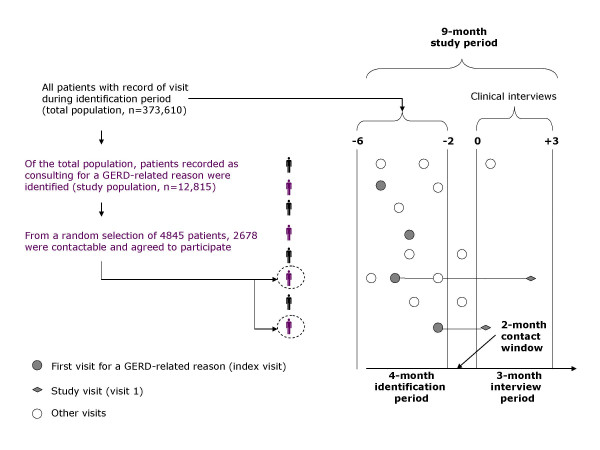
**Study design and patient flow**. GERD, gastroesophageal reflux disease.

The full design of the RANGE study is illustrated in Figure 1. During a 4-month identification period, all subjects (aged ≥ 18 years) who consulted with their PCP for any reason were identified ('total population'). Based on a retrospective medical record review of the total population, patients consulting at least once for a GERD-related reason (with or without treatment, and regardless of whether GERD was the main reason for the visit) were identified ('study population'). Subjects were considered to have consulted their PCP for a GERD-related reason if they met at least one of the following criteria: they reported troublesome heartburn and/or regurgitation; GERD had been diagnosed by endoscopy (presence of esophagitis), esophageal pH monitoring (pathological esophageal pH) or by the presence of symptoms only (heartburn and/or regurgitation); GERD complications were recorded (including haemorrhage, stricture or Barrett's metaplasia); or they were prescribed acid-suppressive medication (proton pump inhibitors [PPIs] or H_2 _receptor antagonists) and/or antacids for GERD. Subjects were included in the study population irrespective of the number of times they had attended the primary care clinic during the identification period. Patients participating in the study were required to provide informed consent. Exclusion criteria were as follows: prophylactic PPI use to prevent ulcers in patients taking non-steroidal anti-inflammatory drugs (NSAIDs); PPI use to heal an NSAID-induced ulcer; PPI treatment for *Helicobacter pylori *eradication; and participation in another clinical study.

From the study population, a randomly selected sample ('selected population') was invited to participate in the study by means of a letter or telephone call. Selection of participants was made using the random number generating function of Microsoft^® ^Office Excel^®^. Those who agreed to participate were asked to attend a clinic visit at which the following data were collected during interview with the PCP and from the subject's medical records: demographics, medical history, reason for initial consultation (e.g. new symptoms in patients who had never previously experienced GERD symptoms, recurrent or persistent symptoms, follow-up visit in an asymptomatic patient), GERD symptoms during the previous 7 days (type, frequency and intensity), diagnostic procedures utilised, GERD complications, and treatment. Patients were asked to complete three self-administered PRO instruments: the Reflux Disease Questionnaire (RDQ) [10] the GERD Impact Scale (GIS) [11] and an extra-esophageal symptoms questionnaire (XQS). The RDQ is a validated 12-item questionnaire designed to assess the frequency and severity of heartburn, regurgitation and dyspeptic symptoms. Items are scored on a 6-point Likert-type scale (range 0–5), with higher scores indicating more severe and/or frequent symptoms. GIS is an easy-to-use tool in which patients grade a number of items (acid-related symptoms, chest pain, extra-esophageal symptoms, the impact of symptoms on sleep, work, meals and social occasions) according to frequency on a 4-point scale (daily = 1, often = 2, sometimes = 3 or never = 4). The XQS is an exploratory, non-validated questionnaire designed to assess the frequency and intensity of sleep disturbance, chest pain, daytime cough, night-time cough, hoarseness, wheezing, difficulty swallowing and nausea on a 6-point Likert-type scale (range 0–5), with higher scores indicating severe or more frequent symptoms (see Additional file 1). Self-evaluation of sleep was also made using relevant items from the Quality of Life in Reflux and Dyspepsia (QOLRAD) questionnaire (night sleep, tired due to lack of sleep, wake up at night, fresh and rested, trouble getting to sleep), which are scored on a 7-point Likert-type scale for which lower scores indicate an increased level of distress and frequency of the problem [12].

### Statistical analysis

Hypothesis testing was not carried out in this study, as the objectives were descriptive. In each country, predefined sample sizes for the randomly selected patients were based on the need to allow comparisons between countries and to reduce the sampling error to below 5%. Predefined sample size for Germany, Greece, Norway and Spain was 500 patients (allowing two-sided 95% confidence intervals to be obtained for single proportions using the large sample normal approximation that will extend 4.4% from the observed proportion for an expected proportion of 50% [the worst possible case]). In the same way, predefined sample size for Sweden and UK was 300 patients (allowing to obtain confidence intervals that will extend 5.7% in the worst possible case).

## Results

### Patients

The total population comprised 373,610 patients (Figure 1). Of these, 12,815 (3.4%) were recorded as consulting for GERD-related reasons and were included in the study population. The selected population (patients who met the eligibility criteria and were randomly selected) included 4845 patients. Of these, 2678 patients (55%) were contactable and attended for consultation at 134 centres; the remainder were either non-contactable (n = 612), non-attendees (n = 196) or declined participation (n = 340), while 1019 patients were not invited on the basis that required by-country samples sizes were reached.

Among participating patients, demographics were generally similar across countries; mean age was around 57 years and 50–60% of patients were female (Table 1). Recurrence of GERD symptoms after a period of remission was the most common reason for the initial visit in Germany (52% of patients), Greece (42%), Norway (33.5%) and the UK (30.5%); in Spain and Sweden, it was for follow-up of an asymptomatic patient (43% and 29%, respectively). Prior to the index visit, a total of 73% of patients were receiving medication for GERD symptoms, ranging from 52% in Greece to 81% in Germany; 56% of the overall patient population were receiving prescription PPIs, although a wide range was observed across the European countries surveyed (19% in Greece to 74% in Germany).

**Table 1:** Demographics of participating patients with gastroesophageal reflux disease, stratified by country of residence

	Germany (n = 495)	Greece (n = 505)	Norway (n = 525)	Spain (n = 477)	Sweden (n = 368)	UK (n = 308)
Females, n (%)	295 (59.6)	265 (52.5)	303 (57.71)	280 (58.7)	223 (60.6)	171 (55.5)
Mean age, years (SD)	58.6 (14.5)	52.5 (14.3)	57.2 (15.2)	59.8 (15.7)	56.2 (15.0)	56.4 (15.5)
Mean weight, kg (SD)	79.0 (15.8)	78.4 (14.5)	78.9 (16.4)	73.8 (13.4)	80.0 (15.4)	78.6 (17.0)

SD = standard deviation.

### Frequency and intensity of GERD symptoms

**Figure 2:**
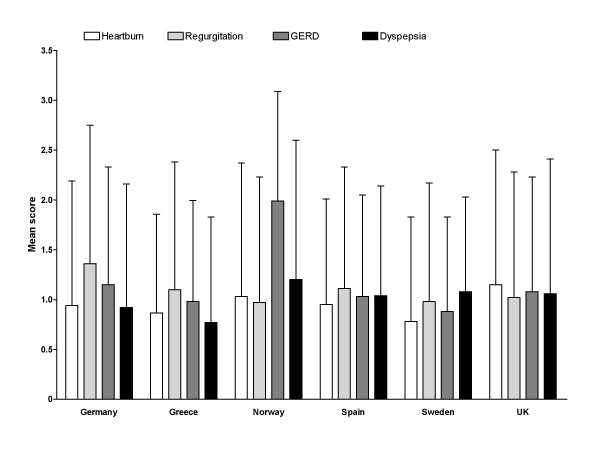
**Mean (standard deviation) Reflux Disease Questionnaire scores, by country of residence**. Scores range from 0 to 5, with higher scores indicating more frequent and/or severe symptoms.

Mean scores for the RDQ dimensions of Heartburn, Regurgitation and Dyspepsia scores (range 0–5, where high score = more severe and/or frequent symptoms) are shown in Figure 2. Among the different countries, mean Heartburn dimension scores ranged from 0.78 (Sweden) to 1.15 (UK), Regurgitation dimension scores ranged from 0.97 (Norway) to 1.36 (Germany), and Dyspepsia dimension scores ranged from 0.77 (Greece) to 1.2 (Norway). While the mean RDQ scores do not indicate a substantial impairment, the data were subject to large standard deviation indicating that at least a portion of the study population experienced more frequent and/or severe symptoms.

### Impact of GERD on daily life

**Figure 3:**
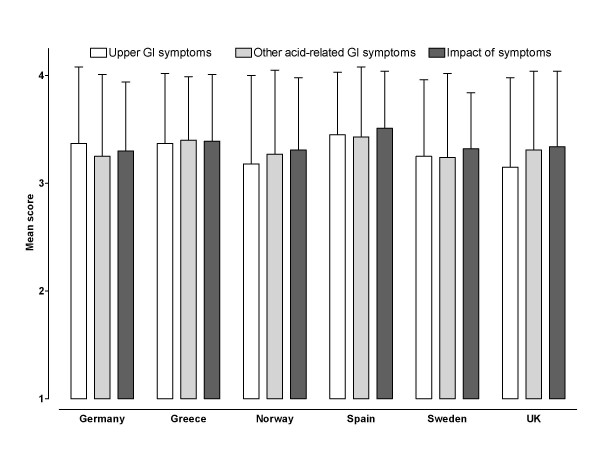
**Mean (standard deviation) GERD Impact Scale scores, by country of residence**. Scores range from 1 to 4, with lower scores indicating increased frequency/impact of symptoms. GI, gastrointestinal.

As shown in Figure 3, mean GIS scores (range 1–4, where low score = worse status) ranged from 3.15 (UK) to 3.45 (Spain) for upper gastrointestinal symptoms and from 3.25 (Germany) to 3.43 (Spain) for other acid-related gastrointestinal symptoms. The mean overall impact score ranged from 3.30 (Germany) to 3.51 (Spain).

**Figure 4:**
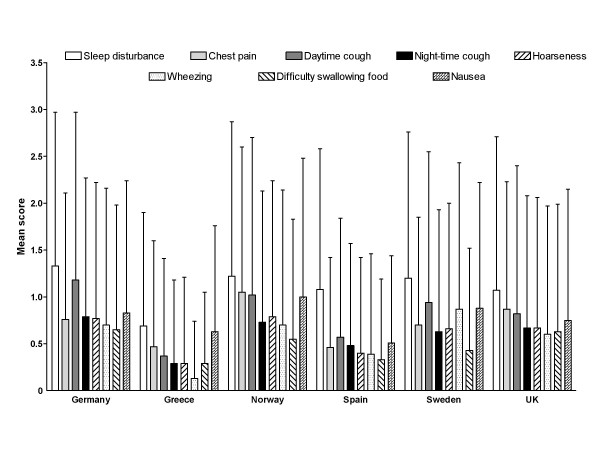
**Mean (standard deviation) Extra-esophageal Symptoms Questionnaire frequency scores, by country of residence**. Scores range from 0 to 5, with higher scores indicating more frequent symptoms.

**Figure 5:**
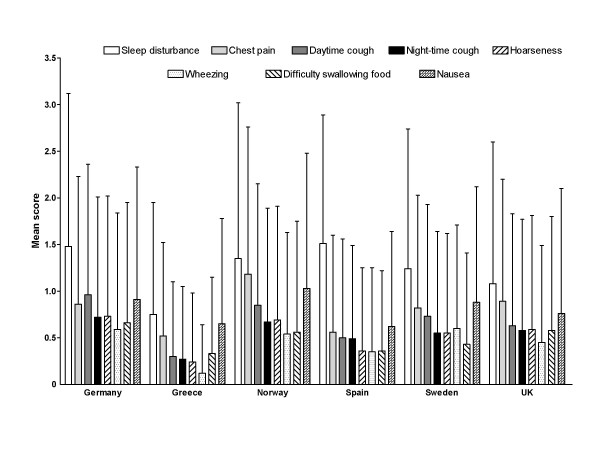
**Mean (standard deviation) Extra-esophageal Symptoms Questionnaire intensity scores, by country of residence**. Scores range from 0 to 5, with higher scores indicating more severe symptoms.

Among extra-esophageal symptoms evaluated using XQS, sleep disturbance scores were consistently higher than scores for other extra-esophageal symptoms across the range of countries in terms of both frequency and intensity (Figures 4 and 5). Similarly to RDQ scores, the XQS data were subject to large variation signifying that there were at least some patients included in this dataset who were affected by extra-esophageal symptoms to a greater extent than others.

Across countries, mean QOLRAD sleep item scores were consistently clustered around 6.0 (data not shown).

## Discussion

This European observational study shows that the heterogeneous population of primary care patients who seek medical attention for GERD continue to experience substantial impairment of their daily lives, as shown by RDQ and GIS scores. Indeed, the combined use of the questionnaires provided a comprehensive overview of the frequency, intensity and impact of GERD symptoms on patients' daily lives, aspects that would not necessarily have been captured by use of one questionnaire alone. Thus, RDQ allowed for an evaluation of the frequency and intensity of GERD symptoms, the GIS providing complimentary information in terms of the use of additional medication for GERD symptoms and the impact of such symptoms on work and daily productivity, eating/drinking and sleep. An association between GERD and sleep disturbance was apparent, as reflected by QOLRAD sleep item scores, XQS scores show that sleep disturbance has more impact on the daily lives of GERD patients than atypical GERD-related symptoms such as cough, hoarseness, wheezing and difficulty swallowing food.

Our findings correlated positively with previous data regarding the impact of symptoms of GERD on the daily lives of patients in European countries [5-7]. It is now particularly apparent that impairment of HRQOL is correlated with patient-perceived severity and frequency of GERD symptoms, and that occurrence of mild but troublesome GERD symptoms at least once a week is a useful indication of underlying GERD [6,7]. Nocturnal symptoms are common in patients with GERD [13,14], and our findings are consistent with the impact of GERD on sleep as previously reported [13-15].

Overall, the findings of the RANGE program highlight the inability of medical therapy to sufficiently control this disease in every case. Effective medical management of GERD relies upon the consulting PCP being able to diagnose and prescribe the most appropriate treatment, and subsequently ensuring that treatment provides sustained relief from symptoms and normalisation of HRQOL [4]. The effectiveness of PPI therapy for improving HRQOL has been demonstrated across the spectrum of patients with GERD in the ProGERD study [5], and evidence-based guidelines recommend PPIs as the most effective first-line treatment approach for patients with GERD [16,17]. Despite such previous findings, it was apparent in the present study that some patients continued to experience symptoms and resulting impact despite prescription therapy (including PPIs). This may, however, be due to the fact that we did not use a common treatment or maintenance protocol, and that patients' compliance with treatment was not assessed.

It is important to note that the RANGE findings are a real-life representation of primary care management of patients who actively consulted their PCP within the previous 6 months. While the results of controlled clinical trials in patients with GERD may show impressive results, the results of the RANGE study support an unmet need for improved management of GERD in the primary care setting. Achieving this objective may be facilitated by improved communication between patients and their PCPs. It is, for example, possible that patients with residual complaints despite having been prescribed continuous PPI treatment on diagnosis of GERD may only be taking their medication on-demand in response to symptoms, or when they anticipate that their symptoms will occur [8]. It is important that such information is effectively obtained from patients, and that patients receive advice regarding the most effective use of different medications for GERD. In those patients whose symptoms are still having a negative impact on their well-being despite good compliance with PPI therapy, it is quite possible that persistent symptoms may be caused by a problem other than acid reflux and that the diagnosis should be reconsidered [14]. However, previous research has confirmed that even adequate therapy with PPIs does not always lead to complete resolution of all GERD-related symptoms, and that there are significant differences between the different PPIs in terms of effectiveness [18]. In this regard, PRO instruments may prove useful in selecting a patient's individualised treatment.

Given the fact that our study population was confined to patients who had consulted a PCP for GERD-related reasons, and that patients generally consult a PCP for GERD because symptoms are having a negative effect on the HRQOL [8], an adverse impact of GERD on the daily lives of patients agreeing to participate in our study was to be expected. While the mean PRO scores may not reflect this, the wide spread of the data (as indicated by large SD values) show that there was a proportion of patients who experienced a high impact due to GERD. The retrospective selection of subjects was designed to obtain a fair representation of the population seeking medical attention because of upper gastrointestinal symptoms related to GERD, as all patients visiting their primary care practitioner within the specified identification period were included in the study population and irrespective of the number of times they visited that practitioner. Also, the high rate of acceptance among the different countries indicates that selection bias should not have had undue influence on the final results, as patients were randomly selected and then invited to participate in the trial.

There are two types of PRO that can be used to measure HRQOL: generic and disease-specific. Generic instruments are designed to evaluate functional status and well-being in general populations, whereas disease-specific instruments focus only on problems relevant to the disease in question. In our study, we utilised PROs that focus on gastrointestinal symptoms of relevance to GERD. One limitation of disease-related instruments is that they may not discriminate between similar diseases [19]. It is, for example, difficult to discriminate between GERD symptoms and similar dyspeptic symptoms that are not a result of acid reflux. While existing PRO instruments do appear to be beneficial in terms of quantifying GERD symptom load and the burden of disease, there is still a need for new reliable and responsive tools that are valid in different languages for international use in the assessment of disease burden in patients with GERD [20]. The new GerdQ questionnaire [21], which combines validated questions from several PRO questionnaires, including the RDQ, GIS and the Gastrointestinal Symptoms Rating Scale, may be one such instrument, providing more accurate and sensitive quantification of the symptoms and the impact of these in patients with GERD, and thereby facilitating better management of disease.

## Conclusion

The findings of this European observational study show that a proportion of patients with GERD are inadequately treated, having clinically relevant impact on their daily lives. These data indicate a need for an improved approach to GERD management in the primary care setting, tailoring treatment on an individual basis in order to lessen the impact of the disease. This may be aided with the use of well suited, validated PRO instruments.

## Abbreviations

GERD: gastroesophageal reflux disease; GIS: GERD Impact Scale; HRQOL: health-related quality of life; PCP: primary care physician; PPI: proton pump inhibitor; PRO: patient-reported outcomes; QOLRAD: Quality of Life in Reflux and Dyspepsia; RDQ: Reflux Disease Questionnaire; XQS: extra-esophageal symptom questionnaire.

## Competing interests

Dr J. P. Gisbert has received educational/research grants and consulting fees from AstraZeneca; Dr A. Cooper has no competing interests to declare; Dr D. Karagiannis has received research grants from Abbott and speaker fees from Janssen, AstraZeneca and Falk (Galenica); Dr J. Hatlebakk has received speaker fees from AstraZeneca; Dr L. Agréus has received research grants and speaker fees from AstraZeneca, and is a former advisory board member for Orexo AB; Dr H. Jablonowski has received speaker fees from AstraZeneca; Dr J. Zapardiel is an employee of AstraZeneca.

## Authors' contributions

All authors were involved in data interpretation and manuscript preparation. Data analysis was provided by AstraZeneca. All authors read and approved the final submission.

## Supplementary Material

Additional file 1 **Extra-esophageal Symptoms Questionnaire**. Summary of the questions (and possible responses) that comprised the Extra-esophageal Symptoms Questionnaire. Click here for file
