# Supplementary material for: Impact of gastroesophageal reflux disease on patients' daily lives: a European observational study in the primary care setting
Source: Health Qual Life Outcomes. 2009 Jul 2;7:60. doi: 10.1186/1477-7525-7-60 (PMC2714504; doi:10.1186/1477-7525-7-60)
Supplement: Additional file 1 — Extra-esophageal Symptoms Questionnaire. Summary of the questions (and possible responses) that comprised the Extra-esophageal Symptoms Questionnaire. [file 1477-7525-7-60-S1.doc]

| Extra-esophageal Symptoms Questionnaire | | | | | | | | | | | | | | | |
| --- | --- | --- | --- | --- | --- | --- | --- | --- | --- | --- | --- | --- | --- | --- | --- |
| *Please answer each question by ticking* ***one*** *box per row.* | | | | | | | | | | | | | | | |
| **Thinking about symptoms over the past 7 days, how would you rate the following?** | | | | | | | | | | | | | | | |
|  | | Did not have | |  | 1 day |  | 2 days |  | 3-4 days | |  | | 5-6 days |  | Daily |
| a. | Sleep disturbance | 🞏 | |  | 🞏 |  | 🞏 |  | 🞏 | |  | | 🞏 |  | 🞏 |
| b. | Chest pain | 🞏 | |  | 🞏 |  | 🞏 |  | 🞏 | |  | | 🞏 |  | 🞏 |
| c. | Daytime cough | 🞏 | |  | 🞏 |  | 🞏 |  | 🞏 | |  | | 🞏 |  | 🞏 |
| d. | Night-time cough | 🞏 | |  | 🞏 |  | 🞏 |  | 🞏 | |  | | 🞏 |  | 🞏 |
| e. | Hoarseness | 🞏 | |  | 🞏 |  | 🞏 |  | 🞏 | |  | | 🞏 |  | 🞏 |
| f. | Wheezing | 🞏 | |  | 🞏 |  | 🞏 |  | 🞏 | |  | | 🞏 |  | 🞏 |
| g. | Difficulty swallowing food through the gullet | 🞏 | |  | 🞏 |  | 🞏 |  | 🞏 | |  | | 🞏 |  | 🞏 |
| h. | Nausea | 🞏 | |  | 🞏 |  | 🞏 |  | 🞏 | |  | | 🞏 |  | 🞏 |
| Thinking about symptoms over the past 7 days, how would you rate the following? | | | | | | | | | | | | | | | |
|  | | | Did not have |  | Very mild |  | Mild |  | Moderate |  | | Moderately  severe | |  | Severe |
| a. | Sleep disturbance | | 🞏 |  | 🞏 |  | 🞏 |  | 🞏 |  | | 🞏 | |  | 🞏 |
| b. | Chest pain | | 🞏 |  | 🞏 |  | 🞏 |  | 🞏 |  | | 🞏 | |  | 🞏 |
| c. | Daytime cough | | 🞏 |  | 🞏 |  | 🞏 |  | 🞏 |  | | 🞏 | |  | 🞏 |
| d. | Night-time cough | | 🞏 |  | 🞏 |  | 🞏 |  | 🞏 |  | | 🞏 | |  | 🞏 |
| e. | Hoarseness | | 🞏 |  | 🞏 |  | 🞏 |  | 🞏 |  | | 🞏 | |  | 🞏 |
| f. | Wheezing | | 🞏 |  | 🞏 |  | 🞏 |  | 🞏 |  | | 🞏 | |  | 🞏 |
| g. | Difficulty swallowing food through the gullet | | 🞏 |  | 🞏 |  | 🞏 |  | 🞏 |  | | 🞏 | |  | 🞏 |
| h. | Nausea | | 🞏 |  | 🞏 |  | 🞏 |  | 🞏 |  | | 🞏 | |  | 🞏 |
